# The Biostimulant, Potassium Humate Ameliorates Abiotic Stress in *Arabidopsis thaliana* by Increasing Starch Availability

**DOI:** 10.3390/ijms241512140

**Published:** 2023-07-28

**Authors:** Patricia Benito, Javier Bellón, Rosa Porcel, Lynne Yenush, José M. Mulet

**Affiliations:** 1 Instituto de Biología Molecular y Celular de Plantas (IBMCP), Universitat Politècnica de València-Consejo Superior de Investigaciones Científicas, 46022 Valencia, Spain; p.benito@caldic.com (P.B.); roporrol@upv.es (R.P.); lynne@ibmcp.upv.es (L.Y.); 2 Caldic Ibérica, S. L. U. Llobateras 23-25, pol.ind. Santiga, Barberà del Vallés, 08210 Barcelona, Spain; javier.bellon@caldic.com

**Keywords:** organic agriculture, metabolomics, agricultural inputs, maltose, proline, salinity, drought, energetic status

## Abstract

Potassium humate is a widely used biostimulant known for its ability to enhance growth and improve tolerance to abiotic stress. However, the molecular mechanisms explaining its effects remain poorly understood. In this study, we investigated the mechanism of action of potassium humate using the model plant *Arabidopsis thaliana*. We demonstrated that a formulation of potassium humate effectively increased the fresh weight accumulation of Arabidopsis plants under normal conditions, salt stress (sodium or lithium chloride), and particularly under osmotic stress (mannitol). Interestingly, plants treated with potassium humate exhibited a reduced antioxidant response and lower proline accumulation, while maintaining photosynthetic activity under stress conditions. The observed sodium and osmotic tolerance induced by humate was not accompanied by increased potassium accumulation. Additionally, metabolomic analysis revealed that potassium humate increased maltose levels under control conditions but decreased levels of fructose. However, under stress, both maltose and glucose levels decreased, suggesting changes in starch utilization and an increase in glycolysis. Starch concentration measurements in leaves showed that plants treated with potassium humate accumulated less starch under control conditions, while under stress, they accumulated starch to levels similar to or higher than control plants. Taken together, our findings suggest that the molecular mechanism underlying the abiotic stress tolerance conferred by potassium humate involves its ability to alter starch content under normal growth conditions and under salt or osmotic stress.

## 1. Introduction

Most agricultural lands face different environmental stresses. Soil salinization and drought are two of the main abiotic factors that affect plant development and decrease crop productivity and quality in most agricultural land worldwide [1]. Abiotic stress also poses a great threat to natural environments [2]. According to the UN (United Nations), desertification, which is the degradation of fertile soil, causes the loss of about 12 million hectares of arable land each year, and the estimations are that over 250 million people are directly affected by desertification [3,4]. UN data indicate that the number and duration of droughts have increased by 29% since the year 2000, while approximately 1.5 Mha were eventually lost due to salinization [5]. Therefore, agricultural production must contend with the fact that these ecological stresses are likely to affect more than three-quarters of the world’s population by 2050. In addition, increasing prices of energy and the high environmental impact pose a serious limitation for the use of fertilizers and pesticides.

Despite the advances in breeding and the development of new varieties and cultivars with increased capacity for nutrient uptake and/or resistance to biotic and abiotic stress [6,7,8], there is a high demand for novel tools, not only at the genetic or the engineering level but also to cope with the new scenario imposed by the anthropogenic global warming. In the last three decades, new agricultural inputs have emerged on the market aimed at enhancing the sustainability and resilience of agricultural production systems through a significant reduction of synthetic agrochemicals such as pesticides and fertilizers [9]. Among these new inputs, biostimulants have gained major interest. Plant biostimulants are products of natural origin that contain one or more substances and/or microorganisms whose function, when applied in small amounts to crops or the rhizosphere, is to stimulate natural processes to improve/benefit nutrient uptake, nutrient efficiency, increase plant tolerance to abiotic stress and/or improve crop quality [10,11,12]. Plant biostimulants are not considered to be fertilizers or pesticides since they do not provide nutrients directly, but rather stimulate the physiological processes of the plant itself to improve the availability and absorption of nutrients. In addition, they do not have any direct action against pests or diseases, otherwise, they would fall into the category of phytosanitary products [10,13]. Biostimulants contain active substances that may be able to sustainably increase or stimulate plant growth and provide plant protection against environmental stress, such as drought and salinity [13].

Humic substances are one of the ten categories of biostimulants currently marketed [10]. Humic substances are diverse types of organic molecules that are formed during microbial and chemical degradation of organic matter in soils [14,15,16]. These substances participate in soil fertilization due to their ability to retain water and nutrients, as well as to enhance the cationic exchange capacity of the soil and promote micronutrient and macronutrient bioavailability. They also present hormone-like activity and improve the soil structure [17,18,19]. Humic substances are classified into humic acids, fulvic acids, and humin, depending on the solubility and pH of the formulation [20,21]. Potassium humate is a potassium salt of humic acid constituted by a diverse complex of humic substances with high solubility. This potassium salt is widely used by farmers as a plant biostimulant and it can improve the physio-biochemical properties of soil and increase its fertility by increasing the amount of available potassium, thus increasing plant yield. Potassium is one of the main macronutrients along with nitrogen and phosphorus that, when used as a fertilizer, increases plant dry matter and enhances productivity [22,23]. In addition, it has been observed that the application of potassium humate relieves abiotic stress because it improves soil health during cultivation [24] and competes with sodium for root absorption [25].

Different authors have observed that the application of humic substances, including potassium humate, affects the production of phytohormones, increases enzymatic and non-enzymatic antioxidant defense, increases the production of osmoprotectants or compatible solutes, such as proline and sugars [26], and changes in the cationic balance, in addition to an indirect effect on plant metabolism [27,28]. This latter effect has been studied through different omic techniques, such as transcriptomics, proteomics, and metabolomics [29,30,31,32,33,34]. Trevisan and colleagues found an upregulation of genes involved in primary metabolism in *Arabidopsis thaliana* plants treated with humic substances [30], while Aguiar et al. [33] observed that the application of humic acids during drought stress in sugarcane significantly decreased the concentration of 15 metabolites, which include amino acids, and they also observed an increase in the levels of 40 compounds related to stress responses (shikimic, caffeic, hydroxycinnamic, valeric and behenic acid, putrescine, quinoline xylulose, galactose, lactose, proline, and oxyproline). Analysis of the effects of humic acids on *Brassica napus* growth showed that several metabolic pathways (such as fatty acids, phytohormones, senescence, plant development, and ion transport) were affected by the application of humic substances [31]. However, despite the available information on the effect of humic substances on the interaction with osmoprotective solutes and the plant metabolome, there are few studies on the specific effect of potassium humate during stress, and its effect at the molecular level. We have previously shown that the model plant *Arabidopsis thaliana* is a good system to evaluate the effect of biostimulants and to investigate the molecular responses elicited by the biostimulant [35,36,37]. The objective of the present study is to gain insights into the molecular mechanism explaining the effect of potassium humate in plants.

## 2. Results

### 2.1. Effect of Potassium Humate (KH60) on the Growth of Arabidopsis thaliana in the Presence of Abiotic Stress

**Figure 1:**
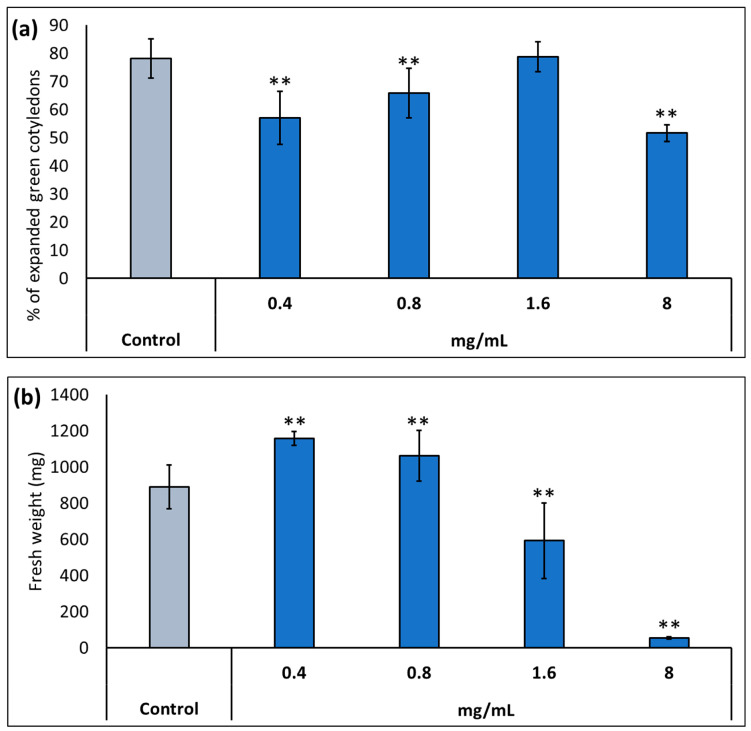
Determination of the optimal dose of potassium humate (KH60) in two stages of *A. thaliana* development: (**a**) complete expansion of the green cotyledons and (**b**) vegetative growth. In the case of vegetative growth, the application of KH60 was carried out 3 days after the transplant, and fresh weight was measured when the plants showed a fully developed rosette stage. The *X*-axis indicates the different concentrations (0.4, 0.8, 1.6, and 8 mg/mL) of the KH60, while the *Y*-axis represents (**a**) the percentage of expanded green cotyledons and (**b**) the fresh weight (mg) of plants grown to full rosette. n = 30 for each individual bar. Bars represent the standard error. ** *p* < 0.01 by Student’s tests for the target sample compared to the control sample.

Throughout this study, we used a commercial formulation of potassium humate provided by Caldic Ibérica S.L.U (see Materials and Methods) named KH60 (Calbio). First, we determined the optimal dose of KH60 under normal conditions in two plant growth stages: germination and early development (the number of germinated seedlings with fully expanded cotyledons was determined), and plants in vegetative growth (fully developed rosette stage). In the case of the percentage of expanded green cotyledons, we observed that the product had a deleterious effect at several of the assayed doses, except at 1.6 mg/mL (Figure 1a). In the vegetative growth phase, the dose with the greatest biostimulant effect was 0.4 mg/mL, while it was toxic at doses of 1.6 mg/mL or higher (Figure 1b).

**Figure 2:**
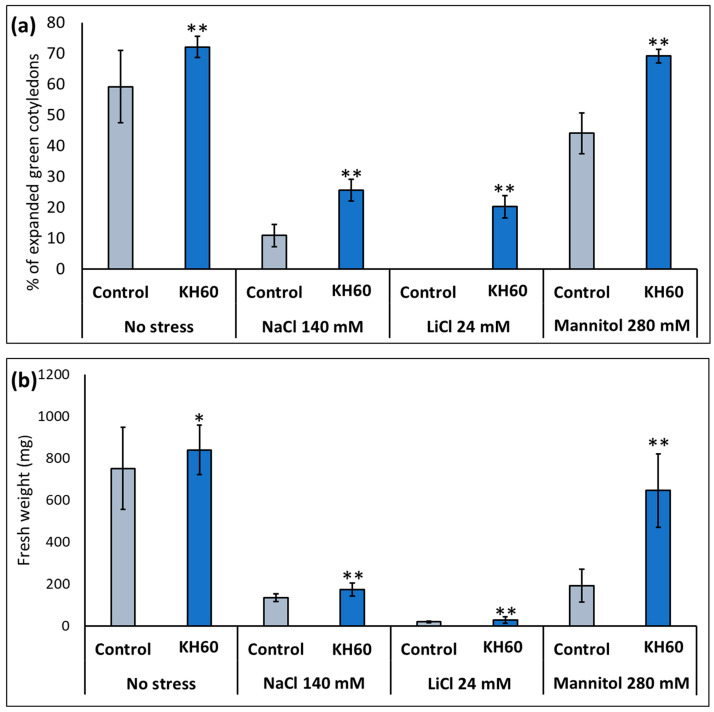
Effect of potassium humate (KH60) in two stages of *A. thaliana* development: (**a**) fully expanded green cotyledons (**b**) vegetative growth under control conditions (no stress), and saline (NaCl and LiCl) and osmotic (mannitol) stress. Optimal KH60 concentration used was 1.6 mg/mL (*w*/*v*) for germination and 0.4 mg/mL (*w*/*v*) for vegetative growth. The application of KH60, during vegetative growth, was carried out 3 days after the transplant, and fresh weight was measured when the plants showed a fully developed rosette stage. The *X*-axis indicates the different concentrations of abiotic stressors. The *Y*-axis represents (**a**) the percentage of expanded green cotyledons and (**b**) the fresh weight (mg) of plants grown to full rosette. n = 30 for each individual bar. Bars represent the standard error. * *p* < 0.05 and ** *p* < 0.01 by Student’s tests for the target sample compared to the control sample under the same conditions.

After determining the optimal concentration, the effect of KH60 was studied under stress conditions. We investigated salt stress (140 mM NaCl or 24 mM LiCl) and osmotic stress (280 mM mannitol). The rationale behind using LiCl is that it is a widely used analog of sodium, but it is toxic at lower concentrations (about 5–6 times lower concentrations); thus, it causes less osmotic stress [25]. Mannitol was used to simulate drought stress as it is an osmophilic molecule that does not enter the plant, but it compromises root water uptake [38]. The presence of KH60 in the medium induced a marked increase in expanded green cotyledons and biomass during vegetative growth under control and under abiotic stress conditions when compared to the control plants under stress (Figure 2). This stimulating effect was especially dramatic for fully opened cotyledons under LiCl stress (1923%) (Figure 2a) and in vegetative growth in the presence of mannitol (239% increase), in this latter case, the plants had a % of expanded green cotyledons and fresh weight similar to an unstressed plant (Figure 2b).

### 2.2. Photosynthetic Pigment Content and Photosystem II Yield Index of Arabidopsis Leaves

**Figure 3:**
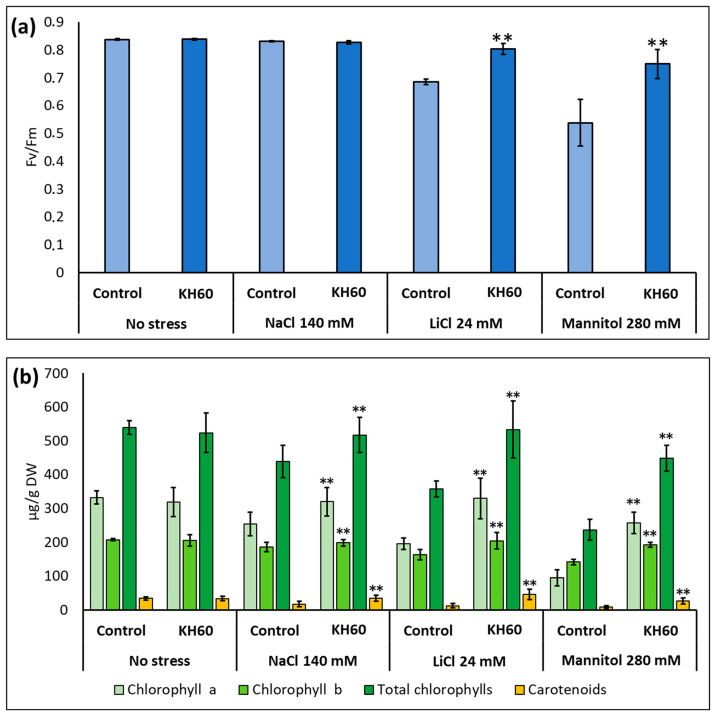
Measurement of photosynthetic activity. (**a**) Photosynthetic yield and (**b**) photosynthetic pigments. The *X*-axis indicates the different concentrations of abiotic stressors. The *Y*-axis represents (**a**) the chlorophyll a yield index (Fv/Fm) is presented as a ratio of variable fluorescence (Fv) over the maximum value of fluorescence (Fm)) and (**b**) µg of photosynthetic pigment per gram of dry weight (DW). n = 10 for each individual bar. Bars represent the standard error. ** *p* < 0.01 by Student’s tests for the target sample compared to the control sample.

We determined the effect of KH60 on the quantum yield of photosystem II and the photosynthetic pigments in Arabidopsis plants at the stage of full rosette development. Under control conditions, KH60 had no effect on any of the assayed parameters. At 24 mM LiCl and at 280 mM mannitol, KH60 was able to maintain the levels similarly to unstressed plants while in the untreated samples, the levels dropped about 20–40% (Figure 3a). The concentrations of chlorophyll a, chlorophyll b, and carotenoids also were maintained at levels in the same range as unstressed plants upon KH60 treatments and under all abiotic stress conditions assayed, especially in the case of LiCl and mannitol (Figure 3b).

### 2.3. Effect of KH60 on the Content of Osmolytes and Non-Enzymatic Antioxidants under Abiotic Stress Conditions

**Figure 4:**
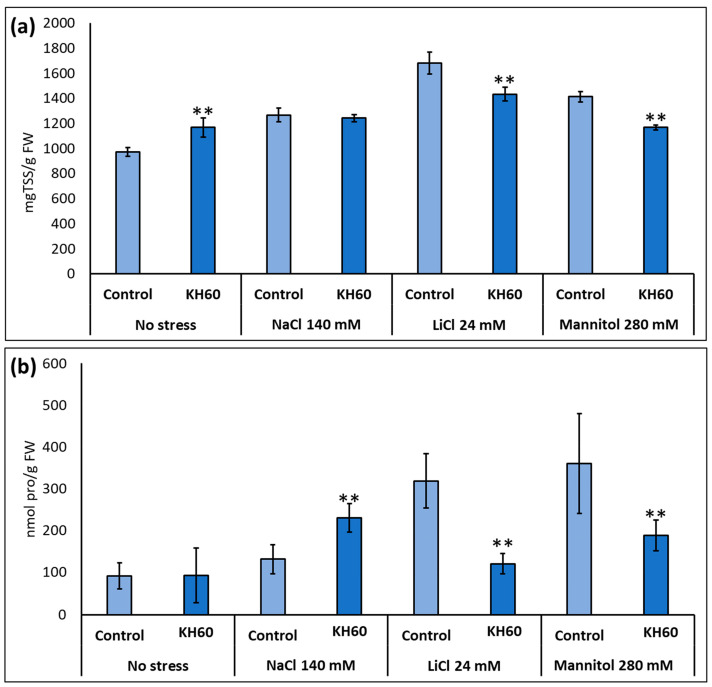
Accumulation of osmolytes under abiotic stress. (**a**) Total soluble sugars (TSS) and (**b**) proline (pro). The *X*-axis indicates the different concentrations of abiotic stressors. The *Y*-axis represents (**a**) mg TSS per gram of fresh weight (FW) and (**b**) nmols of proline per gram of fresh weight (FW). n = 6 for each individual bar. Bars represent the standard error. ** *p* < 0.01 by Student’s tests for the target sample compared to the control sample.

Under salt or drought stress, Arabidopsis accumulates osmolytes like proline or soluble sugars to prevent water loss and maintain turgor. We analyzed the effect of KH60 on osmolyte accumulation during abiotic stress by measuring proline and total soluble sugars. In both cases, a decrease in the concentration of proline and total sugars was observed upon KH60 treatments under LiCl and mannitol stress (Figure 4). In the case of total sugars, there was a decrease of 17% in the presence of LiCl and 21% in the presence of mannitol (Figure 4a). We also observed a decrease in the proline content of 164% for LiCl and 91% for mannitol, as compared to control samples (Figure 4b). 

### 2.4. Effect of Stress and KH60 on Ion Content

**Figure 5:**
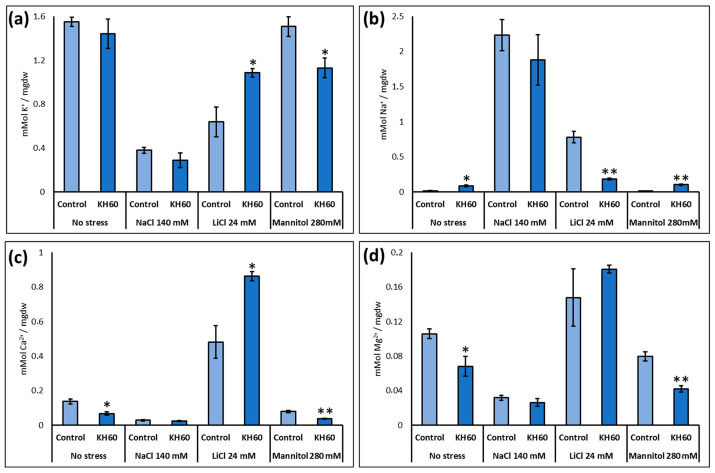
Determination of (**a**) potassium (**b**) sodium (**c**) calcium and (**d**) magnesium. The *X*-axis indicates the different concentrations of abiotic stressors. The *Y*-axis represents the mMol of the indicated ion per milligram of dry weight (mgdw). n = 6 for each individual bar. Bars represent the standard error. * *p* < 0.05 and ** *p* < 0.01 by Student’s tests for the target sample compared to the control sample.

Because salt and osmotic stress alter plant ion content and potassium may be used by the plant also as an osmolyte to prevent turgor loss, we determined the effect of our product on Na^+^, K^+^, Ca^2+^, and Mg^2+^ content under standard and stress conditions (Figure 5). A decrease in the concentration of Na^+^, K^+^, Ca^2+^, and Mg^2+^ ions was observed in the presence of KH60 under osmotic stress, while the amount of K^+^ and Ca^2+^ increased in plants treated with KH60 in the presence of LiCl.

### 2.5. Effect of KH60 on the Arabidopsis Metabolome under Different Abiotic Stress Conditions

An untargeted metabolomic approach was performed to investigate the metabolic responses of *A. thaliana* treated with potassium humate (KH60) under salt or osmotic stress. The metabolomic analysis was carried out in *A. thaliana* leaves sampled after the complete formation of the rosette. Overall, the method allowed for the identification of 42 primary metabolites. To visualize the compositional variability of the metabolites in the different conditions tested among the different biological replicates, the clean data were normalized using both the internal standard and the sum intensity of the peaks in each sample and then subjected to principal component analysis (PCA). This analysis was performed to have a general perspective of the variability among different replicates of the same sampling. The results indicated that different biological replicates of the same stress or KH60 treatment grouped together, thus validating the experimental design and the sampling procedure (Supplementary Figure S1).

After that, we performed a partial least squares discriminant analysis (PLS-DA), which showed a significant separation and distribution between the two groups under the different conditions tested (with or without humate). This result indicated that the metabolic composition of the *Arabidopsis* leaf changed upon potassium humate application. A grouping of the biological replicates in the same node was observed, both in the analysis of all the samples with each other and upon individual evaluation of each tested condition (Supplementary Figure S2).

**Figure 6:**
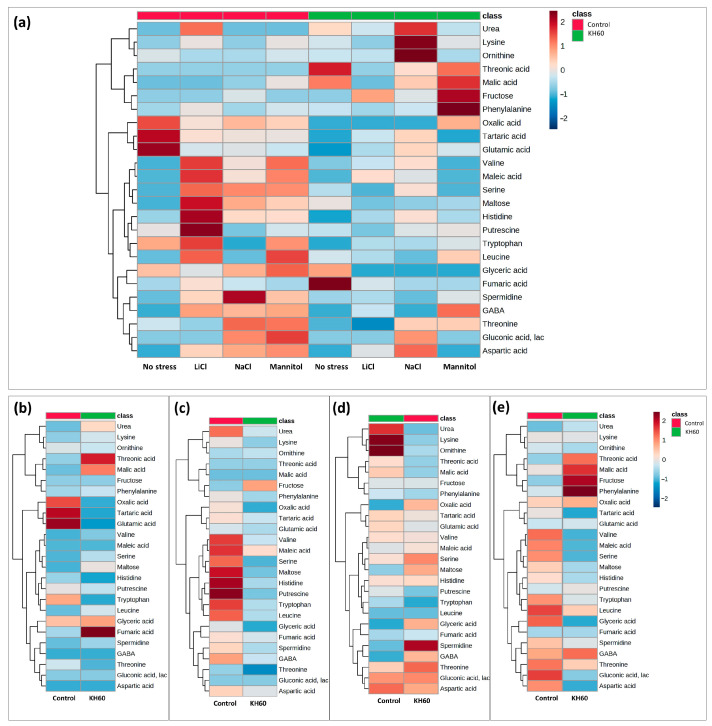
Hierarchical clustering analysis of the correlation between each of the measured *A. thaliana* metabolites and four tested conditions. (**a**) Comparative analysis of the accumulation patterns under the four tested conditions (normal, saline (NaCl and LiCl), and osmotic stress (mannitol)) and presence or absence of potassium humate (KH60). Cluster analysis of accumulated metabolites under (**b**) normal, (**c**) 24 mM LiCl, (**d**) 140 mM NaCl, and (**e**) 280 mM mannitol conditions. Different color scales represent different relative metabolite levels; red indicates up-accumulation and blue indicates down-accumulation of metabolites. n = 4 for each condition.

To monitor the changes for individual metabolites, we performed a hierarchical clustering analysis of the 25 most represented primary metabolites, visually distributing the metabolites into up-accumulated and down-accumulated (Figure 6).

**Figure 7:**
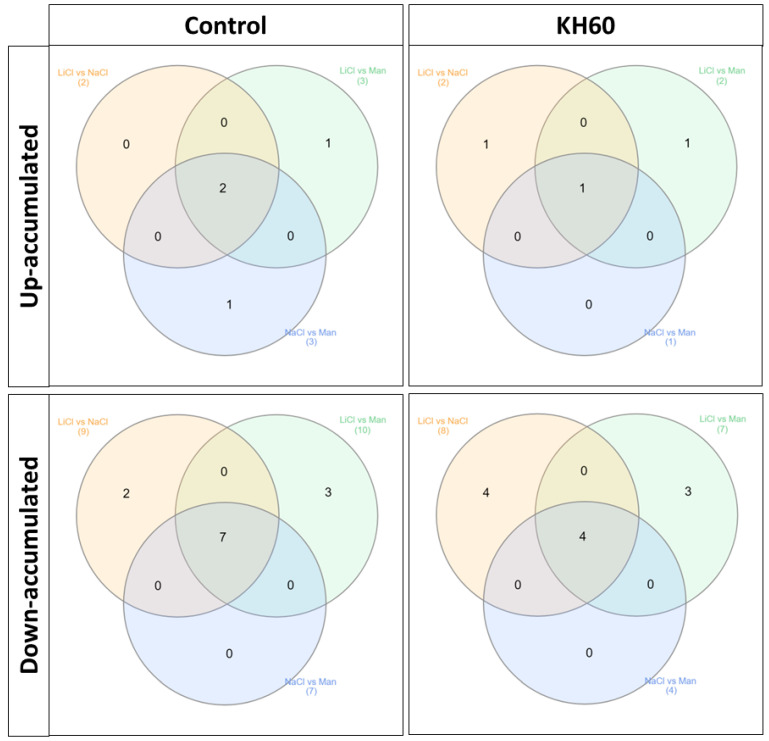
Venn diagrams of common and unique metabolites between common significantly differentially accumulated metabolites after comparing the different abiotic stress conditions: LiCl vs. NaCl, LiCl vs. Mannitol, and NaCl vs. Mannitol. The Venn diagrams correspond to up-accumulated metabolites under control treatment (**upper panel left**), up-accumulated metabolites under KH60 treatment (**upper panel right**), down-accumulated metabolites under control treatment (**lower panel left**), and down-accumulated metabolites under KH60 treatment (**lower panel right**). Numers in parenthesis represent the total amount of metabolits per circle.

To analyze common patterns in different treatments we used Venn diagrams (Figure 7, Table 1). The numbers in the intersections of different circles represent the number of individual metabolites that are significantly accumulated in different stress conditions: LiCl vs. NaCl, LiCl vs. mannitol, NaCl vs. mannitol, or in the three treatments (inner overlapping). This analysis was performed separately in the control and in the KH60 treatment (Figure 7, Table 1). This allowed us to obtain the common differential metabolites between the different tested conditions present in the treatment with and without potassium humate. A table including the comparisons of each stress treatment with respect to the control conditions can be found in the Supplementary material (Supplementary Table S1). It was observed that among up-accumulated metabolites in the control treatment, phosphoric acid and maltose were the two common metabolites in the three comparisons, while glutamine was the only common metabolite among up-accumulated metabolites in the KH60 treatment. In the case of down-accumulated metabolites in the control (no KH60) treatment, it was observed that fructose and six acid metabolites were common in the three stress conditions. In the presence of KH60, the common down-accumulated metabolites under the three stress conditions were fructose, maltose, threonic acid, and glucose.

**Figure 8:**
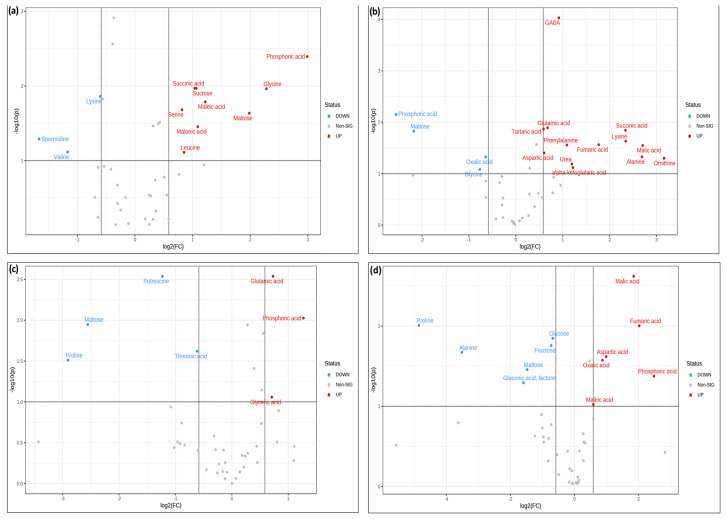
Volcano plot analysis of metabolic changes under (**a**) normal, (**b**) 140 mM NaCl, (**c**) 24 mM LiCl, and (**d**) 280 mM mannitol conditions. A comparison of the samples containing KH60 with respect to the control samples without potassium humate was made to obtain significantly differentially accumulated metabolites. The *X*-axis indicates fold change threshold (FC ≥ 1.5), while the *Y*-axis represented the *t*-test threshold (*p*-value ≤ 0.05). Different colors represent different relative metabolite levels; red indicates significant up-accumulation and blue indicates significant down-accumulation of metabolites. Grey points represent metabolites for which no significant changes were observed.

The statistical significance of these groupings was analyzed by confronting fold change against significance using volcano plots (Figure 8). In these plots, the up-accumulated and down-accumulated metabolites were compared with their significance according to a Student’s test (Figure 8).

**Figure 9:**
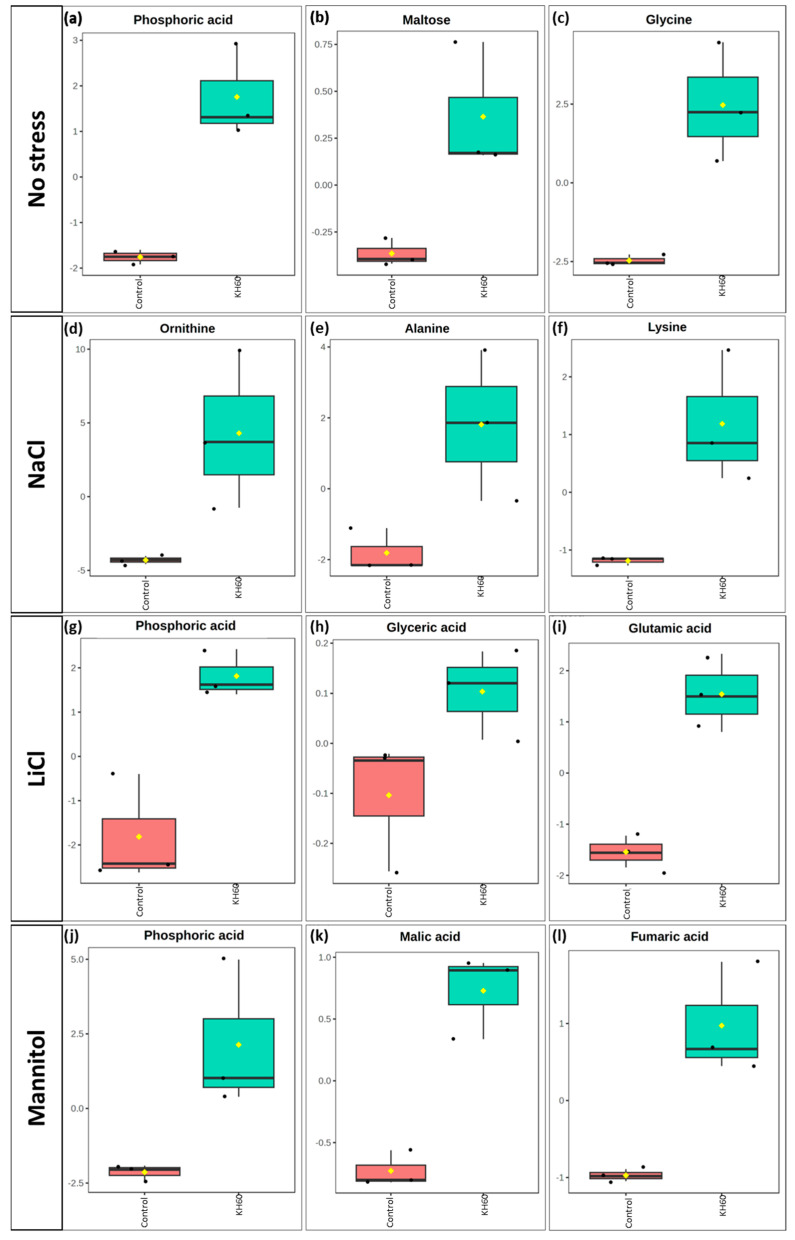
Boxplot of the most significantly over-accumulated metabolites under (**a**–**c**) normal, (**d**–**f**) 140 mM NaCl, (**g**–**i**) 24 mM LiCl, and (**j**–**l**) 280 mM mannitol conditions. The X-axes show the treatment with KH60 next to its control. The Y-axes are represented as relative units. The data were normalized to the total spectral area. Due to this normalization process, a negative scaling on the *Y*-axis was obtained in some cases. The boxes range from the 25% and 75% percentiles; the 5% and 95% percentiles are indicated as error bars; Individual data points are indicated by circles (n = 3). Medians are indicated by horizontal lines inside each box. The mean concentration of each data set is indicated by a yellow diamond.

Further analysis of the over-accumulated and under-accumulated metabolites showed that among the significantly up-accumulated metabolites in the presence of potassium humate, phosphoric acid stood out under normal conditions and under LiCl and mannitol stress (Figure 9a,g,j).

**Figure 10:**
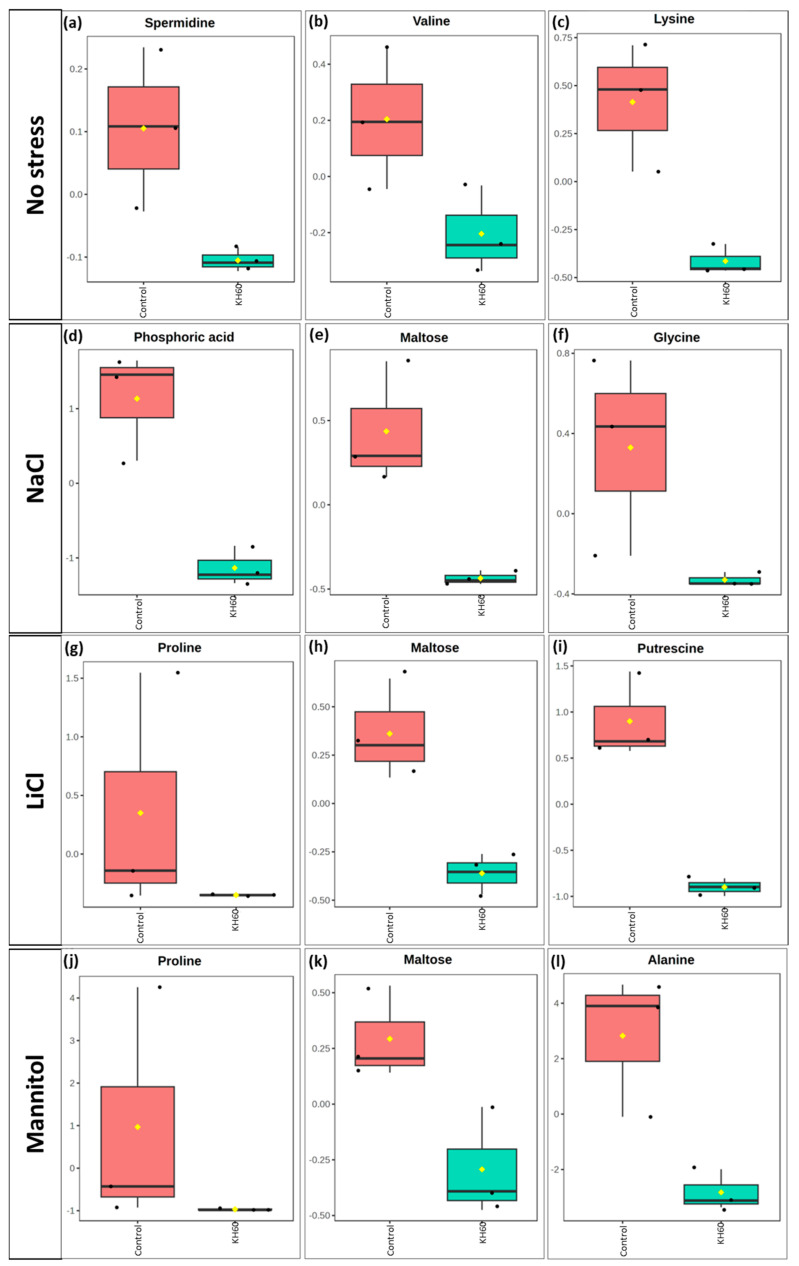
Boxplot of the most significantly down-accumulated metabolites under (**a**–**c**) normal, (**d**–**f**) 140 mM NaCl, (**g**–**i**) 24 mM LiCl, and (**j**–**l**) 280 mM mannitol conditions. The *X*-axis shows the treatment with KH60 next to its control. The Y-axes are represented as relative units. The data were normalized to the total spectral area. Due to this normalization process, a negative scaling on the *Y*-axis was obtained in some cases. Boxes range from the 25% and 75% percentiles; the 5% and 95% percentiles are indicated as error bars; individual data points are indicated by circles (n = 3). Medians are indicated by horizontal lines inside each box. The mean concentration of each data set is indicated by a yellow diamond.

However, among the down-accumulated metabolites, proline stood out under LiCl and mannitol conditions (Figure 10g,j), while maltose was down-accumulated under NaCl, LiCl, and mannitol conditions (Figure 10e,h,k).

### 2.6. Effect of KH60 on Starch Levels

**Figure 11:**
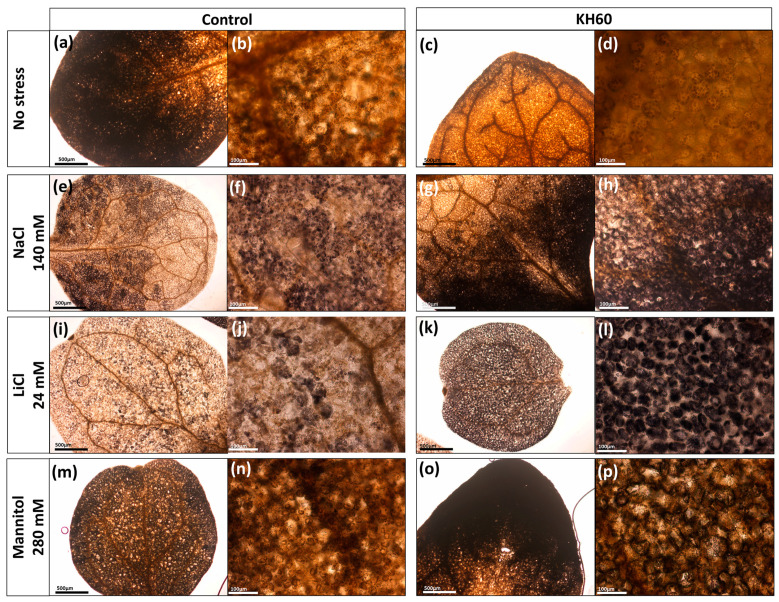
Effect of KH60 on starch accumulation in Arabidopsis leaf cells under (**a**–**d**) normal conditions and abiotic stress conditions exerted by (**e**–**h**) 140 mM NaCl, (**i**–**l**) 24 mM LiCl, and (**m**–**p**) mannitol 280 mM. The plants were grown for 10 days under abiotic stress conditions and in the presence of potassium humate KH60 and stained with Lugol, as described in Materials and Methods (**c**,**d**,**g**,**h**,**k**,**l**,**o**,**p**). Pictures were taken with a stereoscope (**a**,**c**,**e**,**g**,**i**,**k**,**m**,**o**; bar size 500 µm) or with an optical microscope (**b**,**d**,**f**,**h**,**j**,**l**,**n**,**p**; bar size 100 µm). The experiment was repeated in 3–5 plants and representative images of a single leaves for each treatment are shown.

When starch is mobilized, it is degraded in the form of the disaccharide maltose. We have previously observed that maltose levels were up-accumulated upon KH60 addition in control conditions, and we also observed that under stress conditions, maltose levels are down-accumulated. We investigated whether these changes in maltose were indicating changes in the pattern of starch accumulation (Figure 11).

Whereas we observed a decrease in control plants treated with KH60, we observed a marked increase in the intensity of the Lugol staining in leaves of NaCl- and LiCl-stressed plants in the presence of KH60. These results suggest that KH60 improves starch mobilization and/or accumulation under abiotic stress conditions.

## 3. Discussion

Potassium humate is a well-known plant growth promoter obtained from the alkaline extraction of lignite. It is used mainly as a soil conditioner and to increase the efficiency of nitrogen and phosphate fertilizers [24]. It is known that the addition of potassium humate increases growth in wheat [39], aromatic plants [40], and in cotton plant fiber, it increases quality and productivity [41]. Potassium humate has proven to be effective to alleviate both salt stress in the common bean [42] and soybean [43], and arsenic toxicity in rice [44], but descriptions of its effect at the molecular level are scarce. Arabidopsis is a standard model system in plant biochemistry and molecular biology. Its genome, proteome, metabolome, and most of its biochemical pathways have been described. In this study, we have taken advantage of this accumulated knowledge to investigate the molecular mechanisms affected by a formulation of potassium humate (KH60).

We have shown that under normal conditions, KH60 effectively promotes growth at the vegetative stage in Arabidopsis but is rather toxic during germination and early development (Figure 1). Nevertheless, KH60 ameliorates the negative effects of abiotic stress conditions at both stages and is particularly effective under osmotic stress, where the fresh weight of stressed plants treated with KH60 is similar to unstressed plants (Figure 2). When a biostimulant increases plant yield under stress conditions, there are two possibilities to explain the observed phenotypes. The presence of the biostimulant is either alleviating the effect of the stressor or it is enhancing the plant stress response. The addition of KH60 to the medium in the presence of stress was able to maintain the photosynthetic quantum yield and the levels of chlorophyll similar to the control conditions (Figure 3). Interestingly, the accumulation of osmolytes such as proline or soluble sugars was lower in stressed plants treated with KH60 (Figure 4). In our metabolomic analysis, proline was also down-accumulated upon KH60 addition under the same conditions (Table 1 and Figure 7), thus confirming the proline enzymatic measurements shown in Figure 4b. Proline is a well-known osmolyte that accumulates under salt and osmotic stress. As expected, the concentration of proline increases under stress with respect to the unstressed plants, indicating that the plants are indeed responding to stress. However, plants treated with KH60 accumulated less proline in the presence of under LiCl or mannitol, as compared to plants subjected to these stresses in the absence of KH60 (Figure 4b). A tempting hypothesis to explain these results would be that KH60 is not activating the stress response but alleviating the effects of the stress. The fact that the plants are more resistant to stress and, at the molecular level, accumulate less of a pivotal molecule for stress response such as proline, suggests that the plants are perceiving less stress in the presence of KH60.

We investigated whether this observed stress alleviation could be explained by potassium. Plants can use potassium as an osmoprotectant. In addition, under salt stress conditions, potassium counteracts sodium toxicity, as there is a complex interplay between potassium and sodium uptake by plants [45]. As KH60 contains potassium in its formulation, it could be hypothesized that plants experienced less stress due to the increase in potassium in the medium. If this would be the case, we would expect a higher potassium accumulation in KH60-treated plants. Our results indicated that under control conditions or sodium chloride stress, there was no significant difference in the potassium content. We observed an increase under lithium chloride stress, but a decrease under stress induced by mannitol. Therefore, potassium content was not a distinctive feature explaining tolerance (Figure 5a). Under salt stress, the sodium concentration was slightly lower in the KH60-treated plants, but the potassium concentration was also lower. Therefore, the Na^+^/K^+^ ratio, a standard parameter to evaluate sodium toxicity in plants, was almost constant, confirming that plants treated with KH60 were experiencing less stress. The results were very different for lithium. Under this condition, we observed a dramatic change induced by KH60, which led to increased potassium accumulation and decreased sodium content. Therefore, KH60 was blocking sodium entry into the cell or promoting active extrusion, but sodium was not being accumulated in the vacuole or in any other compartment, as we did not observe hyperaccumulation (Figure 5a,b). KH60 also induced higher calcium accumulation under LiCl stress (Figure 5c). Therefore, at least under one of the assayed conditions, KH60 was inducing a change in the mechanisms of ion homeostasis.

To further characterize the molecular effect of KH60, we investigated the changes in the metabolome under the tested conditions. A global view of the common metabolites upon a comparison of the different stress conditions tested showed that phosphoric acid and maltose were accumulated, and several acids (fumaric, malic, threonic, aspartic, glutamic, and tartaric), as well as glucose, were down-accumulated (Figure 7 and Table 1). The KH60 treatment induced a complete change in the metabolic profiles. The only metabolite up-accumulated in the three stress conditions was glutamine, while maltose, fructose, glucose, and threonic acid were down-accumulated upon stress in the presence of KH60 (Figure 7 and Table 1). The most striking result is related to the levels of maltose. Under normal conditions, there is a four-fold increase in the maltose concentration upon KH60 addition, but under stress, maltose concentrations decrease in all the studied stress conditions to a similar level (Figure 8, Figure 9 and Figure 10). Maltose is the main vector for carbon export from chloroplasts at night. Under daylight respiratory conditions, maltose increases in leaves. Maltose metabolism is regulated by, among others, the circadian clock, day length, and temperature. It has been hypothesized that maltose metabolism is crucial for diverting energy from the starch present in leaves to the stress response [46]. From an energetic point of view, stress responses are very costly for the plant [47]. We have previously observed that the accumulation of Krebs cycle intermediates is the distinctive factor among salt-sensitive and salt-tolerant broccoli cultivars [7]. We confirmed that KH60 was indeed altering the pattern of starch accumulation in Arabidopsis. Under control conditions, KH60-treated plants do not accumulate more starch, while upon stress, KH60 induced a higher accumulation of starch, in comparison to stressed plants without KH60 (Figure 11). Starch is considered a determinant of plant fitness under abiotic stress [48].

Starch is synthesized in chloroplasts in the presence of light and used mainly during the night. This provides a supply of carbon and energy in the absence of photosynthetic activity. This process has been studied in Arabidopsis, and most enzymes participating in this complex regulatory process have been identified [49]. In addition to its role as a reservoir of energy, starch has a pivotal role under abiotic stress conditions. Activating the stress response signaling pathways is a costly metabolic process. In addition, photosynthesis may be less efficient under stress. Under stress conditions, plants break down starch to provide energy and carbon. This increase in free sugars derived from starch has a dual effect: it increases the amount of available energy and the sugars act as osmolytes to counteract the effect of stress [50]. In most plants, the starch content decreases in response to abiotic stress [48]. It has been reported that the differential trait between a drought-tolerant and a drought-sensitive cultivar of common bean (*Phaseolus vulgaris*) is the ability of the tolerant cultivar to degrade starch [51]. Another study provided additional evidence that the amount of free sugars is a distinctive trait for salt-tolerant cultivars [52] and a drought-resistant cultivar presented elevated accumulation of carbohydrates in the seeds [53]. A recent report also found that, under drought stress, waxy maize alters the pattern and the physicochemical properties of starch [54].

Under normal conditions, KH60 induces the mobilization of starch, and thus, the plant has more available energy in the form of free sugars. This would explain the increase in fresh weight and enhanced early development observed in Figure 1 and Figure 2. We have confirmed this increase in available energy and osmolytes by determining the total free sugar content under control conditions (Figure 4a). Increased sugar availability would be an advantage when plants are under stress conditions and may explain the fact that most stress response indicators evaluated, such as photosynthesis (Figure 3), proline content (Figure 4b), and potassium accumulation (Figure 5a) are lower in KH60-treated plants. These data suggest that the plants treated with the biostimulants are perceiving less stress, probably because of the increased sugar availability that enables a better stress response. Under stress conditions, treated plants have lower total sugar concentrations (Figure 4a), which is in agreement with the observed increase in starch accumulation observed in Figure 11. Therefore, taken together, our data indicate that the effect of KH60 on starch accumulation may be the molecular factor explaining the observed growth-promoting properties and stress tolerance.

## 4. Materials and Methods

### 4.1. Biostimulant Product, Plant Media, and Growth Conditions

The Calbio potassium humate (KH60) product used in this study was provided by Caldic Ibérica S.L.U (Barcelona, Spain). A working solution was prepared at a concentration of 10 mg/mL (*w*/*v*) and sterilized by tyndalization.

Wild-type *Arabidopsis thaliana* seeds (Columbia-0 ecotype) were surface sterilized with commercial bleach diluted 1:1 (*v*/*v*) for 15 min and rinsed with sterile water. Stratification was carried out for three days at 4 °C. The plant growth medium used in the tests was MS medium containing a mixture of Murashige and Skoog (MS) basal salts (0.22%; Duchefa Biochemie B V, Haarlem, The Netherlands), sucrose (1%), and 2.6 mM MES (2-(N-morpholino) ethanesulfonic acid buffer), adjusted to pH 5.9 with potassium hydroxide. In all assays, plants were grown under long-day chamber conditions (16 h light/8 h dark, 23 °C, 130 μE m^−2^ s^−1^, 70% relative humidity). When specified, the medium was supplemented with 140 mM NaCl or 24 mM LiCl for saline stress and 280 mM mannitol for osmotic stress, and/or the biostimulant KH60 depending on the assay.

### 4.2. Arabidopsis Germination and Early Development under Abiotic Stress

First, the optimal dose with biostimulant effect of potassium humate KH60 on germination and early growth of *Arabidopsis* was determined. For this, the effect of different concentrations of KH60 (0.4, 0.8, 1.6, 8, and 20 mg/mL) under normal conditions was evaluated.

For in vitro germination assays, thirty previously surface-sterilized and stratified seeds were plated on each plate with MS medium containing phytoagar and cultured under long-day chamber conditions for 6 days. When indicated, the medium was supplemented with NaCl, LiCl, or mannitol, as indicated in each case, and KH60 according to the assay. Data for green cotyledons expanded under different conditions and were recorded after 6 days. For growth to an adult plant, seedlings previously germinated for 10 days in MS solid medium containing 0.8% phytoagar, without additives, were transferred to previously hydrated 7 mm Jiffy-42. Irrigation was carried out three times per week two with water and one with liquid MS solution (without phytoagar). Three days after the transplant, 1 mL of the KH60 biostimulant was applied per pellet at a concentration of 1.6 mg/mL. The stress was applied 10 days after transplanting the seedlings to the Jiffy pellets in the irrigation solution with the indicated concentration of NaCl, LiCl, and Mannitol until the plants reached the silique stage. The second and third youngest leaves were then collected for further biochemical analysis. All samples were frozen in liquid nitrogen immediately and stored at −80 °C until needed.

### 4.3. Leaf Photosynthetic Pigments and Photosystem II Yield

Leaf pigments, including chlorophylls and carotenoids, were extracted and quantified using the method described by Lichtenthaler et. al. [55]. Fresh Arabidopsis leaves sample (100 mg) was macerated in methanol 100%, followed by shaking for 30 min at room temperature. After the samples were centrifuged, the resulting solution was used to examine two chlorophyll fractions and carotenoids by using a fluorescence multi-plate reader (Infinite 200 PRO; Tecan) and calculated according to the solvent (methanol 100%) used [55]. Chlorophyll a absorbs light at 665.2 nm; chlorophyll b absorbs light at 652.4 nm, while the total chlorophylls were estimated by the sum of chlorophyll a and b. The concentration of total carotenoids (C − x + c) was then estimated by subtracting the relative absorption of chlorophyll a and chlorophyll b from the absorbance reading at 470 nm and dividing by the absorption coefficient of total carotenoids at 470 nm. Three biological and three technical replicates of each treatment were analyzed.

Photosystem II yield indexes were measured with the HandyPEA fluorimeter (Hansatech, Pentney, UK). Before the measurement, the plant leaves were dark adapted for 45 min. Measurements were made on 10 plants per treatment.

### 4.4. Proline and Total Sugars Determination

Free proline and total soluble sugars were extracted from 100 mg of fresh leaves [56]. Briefly, the extraction was performed using methanol, chloroform, and 0.88% NaCl sequentially in a ratio (2:2:1). The reagent amount was adjusted according to the starting plant material. The methanolic phase was used for the quantification of both substances. Proline was estimated from 100 µL of the extract by spectrophotometric analysis at 520 nm of the ninhydrin reaction (1.25 g ninhydrin in 30 mL glacial acetic acid and 20 mL 6 M phosphoric acid), according to Bates et al. [57]. The calibration curve was made using proline in the range of 0–300 μM. Soluble sugars (TSS) were analyzed by 0.1 mL of the methanolic extract reacting with 3 mL freshly prepared anthrone (200 mg anthrone + 100 mL 72% (*v*/*v*) H_2_SO_4_) and placed in a boiling water bath for 10 min according to Irigoyen et al. [58]. After cooling, the absorbance at 620 nm was determined in a fluorescence multi-plate reader (Infinite 200 PRO; Tecan). The calibration curve was made using glucose in the range of 20–400 μg/mL. The results were expressed as µg of TSS per gram of fresh weight (FW) or µg of proline per gram of FW.

### 4.5. Total Phenols and Flavonoid Determination

The analysis of total phenols and flavonoids was carried out from the methanolic phase used in the determination of sugars and proline. The determination of total phenols was made according to Blainski et al. [59] based on the Folin–Ciocalteu colorimetric method. Briefly, the extracts were incubated with commercial Folin–Ciocalteu reagent (Reagecon) for 5 min and 90 min with 15% sodium carbonate in the dark. The absorbance was measured at 765 nm. As a reference standard, gallic acid was used (0–150 μg/mL concentration range). The results were expressed as µg of gallic acid equivalents (GA) per mg of dry weight (DW). The total flavonoid content was determined based on the aluminum chloride colorimetric method [60]. Briefly, 5% sodium nitrite was added to the extracts and incubated for 5 min, followed by incubation with 10% aluminum chloride. After the addition of 1 M sodium hydroxide, the optical density was determined at 510 nm. As a reference standard, catechin was used (0–90 μg/mL concentration range). The results were expressed as µg of catechin equivalents (CAT) per mg of DW.

### 4.6. Ion Content Determination

Ions were determined as described [38]. Briefly, samples of the second youngest *Arabidopsis* rosette leaf were freeze-dried for two days. Dry weight was determined, and ions were extracted by a 30 min incubation in 1 mL of 0.1 M HNO_3_ at room temperature. Then samples were centrifuged, and the supernatant was diluted with 4 mL of Milli-Q water and filtered (0.22 μm). Sodium and potassium were measured in a plasma emission spectrophotometer (Shimadzu, Kyoto, Japan), as described [61]. Measurements were normalized to dry weight. Three biological replicates of each treatment were analyzed.

### 4.7. Staining of Arabidopsis Leaves with Lugol

Lugol staining of leaves makes it possible to visualize the starch content within plant cells [62,63]. First, the leaves were depigmented with 70% ethanol at 37 °C for 24 h. Subsequently, the ethanol was removed, and leaves were washed with distilled water. Staining was performed with Lugol for 10 min at room temperature until a dark brown color was observed. Finally, Lugol was removed and washed with distilled water. The starch content of leaf cells was visualized using light microscopy.

### 4.8. Metabolomic Analysis

The second youngest leaves of the Arabidopsis rosette were collected and lyophilized and then homogenized with a mechanical tissue disrupter in the presence of liquid nitrogen before obtaining 10 mg of sample powder for each replicate. Four biological replicates of each treatment were used. The analysis of primary metabolites was carried out in the Metabolomics Platform of the Institute of Plant Molecular and Cellular Biology (UPV-CSIC, Valencia, Spain) by gas chromatography coupled with mass spectrometry (GC-MS) and using a method modified from that described by Roessner et al. [64]. Chromatograms and mass spectra were evaluated using the CHROMATOF program (LECO, St. Joseph, MI, USA), and the obtained data were analyzed by MetaboAnalyst 5.0 software (Wishart Research Group, University of Alberta, Edmonton, AB, Canada) [65].

### 4.9. Statistical Analysis

The data on the effect of KH60 on *Arabidopsis* growth as well as on the accumulation of osmoprotectants and non-enzymatic antioxidants and photosynthetic activity were processed using R Statistical Software (v4.3.1; R Core Team 2023). Student’s test was calculated by comparing the results obtained for each treatment to the control conditions.

Metabolic data were normalized and processed in MetaboAnalyst 5.0 software (Wishart Research Group, University of Alberta, Edmonton, AB, Canada) [65]. Data normalization was performed by constant sum and Pareto scaling. Metabolites with fold changes > 1.5 and a Student’s test *p*-value < 0.05 were considered statistically significant. Metabolic pathway analysis was then performed via MetaboAnalyst 5.0 to identify the affected metabolic pathways analysis and visualization.

## 5. Conclusions

In conclusion, our study demonstrates the ability to elucidate the molecular mechanisms governing the abiotic stress tolerance mediated by a formulation of potassium humate (KH60) through the use of a model organism such as Arabidopsis. The application of KH60 effectively alleviates the detrimental impact of stress on plants, with the most significant effect observed under osmotic stress. This effectiveness is evidenced by the reduction in stress indicators, including chlorophyll degradation, antioxidant response, proline accumulation, and potassium accumulation. Additionally, our metabolomic analysis reveals that KH60-treated plants display an accumulation of maltose under normal conditions, which is preferentially degraded under stress conditions. These changes in maltose levels correspond with alterations in the pattern of starch accumulation, a known factor contributing to abiotic stress tolerance. Thus, the observed phenotypes can potentially be explained by the modulation of starch accumulation. Overall, these findings shed light on the underlying mechanisms through which KH60 exerts its beneficial effects on plant growth and stress resilience. By providing a comprehensive understanding of the molecular interactions involved, our study contributes to the advancement of knowledge in this field and paves the way for further research and potential applications in enhancing crop productivity and sustainability.

## Figures and Tables

**Table 1:** Identity of the significantly differentially accumulated metabolites among the different conditions assayed, represented in Figure 7.

Conditions	Common Up-Accumulated Metabolites (No KH60)	Conditions	Common Up-Accumulated Metabolites (In the Presence of KH60)
[LiCl vs. Man]	Sucrose	[LiCl vs. NaCl]	Histidine
[NaCl vs. Man]	Serine	[LiCl vs. Man]	Pyroglutamic acid
[LiCl vs. NaCl] [LiCl vs. Man] [NaCl vs. Man]	Phosphoric acid, Maltose	[LiCl vs. NaCl] [LiCl vs. Man] [NaCl vs. Man]	Glutamine
**Conditions**	**Common Down-Accumulated Metabolites (No KH60)**	**Conditions**	**Common Down-Accumulated Metabolites (In the Presence of KH60)**
[LiCl vs. NaCl]	Succinic acid, Asparagine	[LiCl vs. NaCl]	Glycine, Glycerol, Maleic acid, Malic acid
[LiCl vs. Man]	Lysine, Oxalic acid, Arginine	[LiCl vs. Man]	Succinic acid, Glutamic acid, Proline
[LiCl vs. NaCl] [LiCl vs. Man] [NaCl vs. Man]	Fumaric acid, Malic acid, Threonic acid, Aspartic acid, Glutamic acid, Tartaric acid, Fructose	[LiCl vs. NaCl] [LiCl vs. Man] [NaCl vs. Man]	Maltose, Threonic acid, Glucose, Fructose

## Data Availability

All the data presented in this study have been used in the figures.

## Supplementary Materials

The supporting information can be downloaded at: https://www.mdpi.com/article/10.3390/ijms241512140/s1.
