# Supplementary material for: The Biostimulant, Potassium Humate Ameliorates Abiotic Stress in Arabidopsis thaliana by Increasing Starch Availability
Source: Int J Mol Sci. 2023 Jul 28;24(15):12140. doi: 10.3390/ijms241512140 (PMC10418871; doi:10.3390/ijms241512140)
Supplement: Supplementary file 1 [file ijms-24-12140-s001.zip › ijms-2516883-supplementary.pdf]

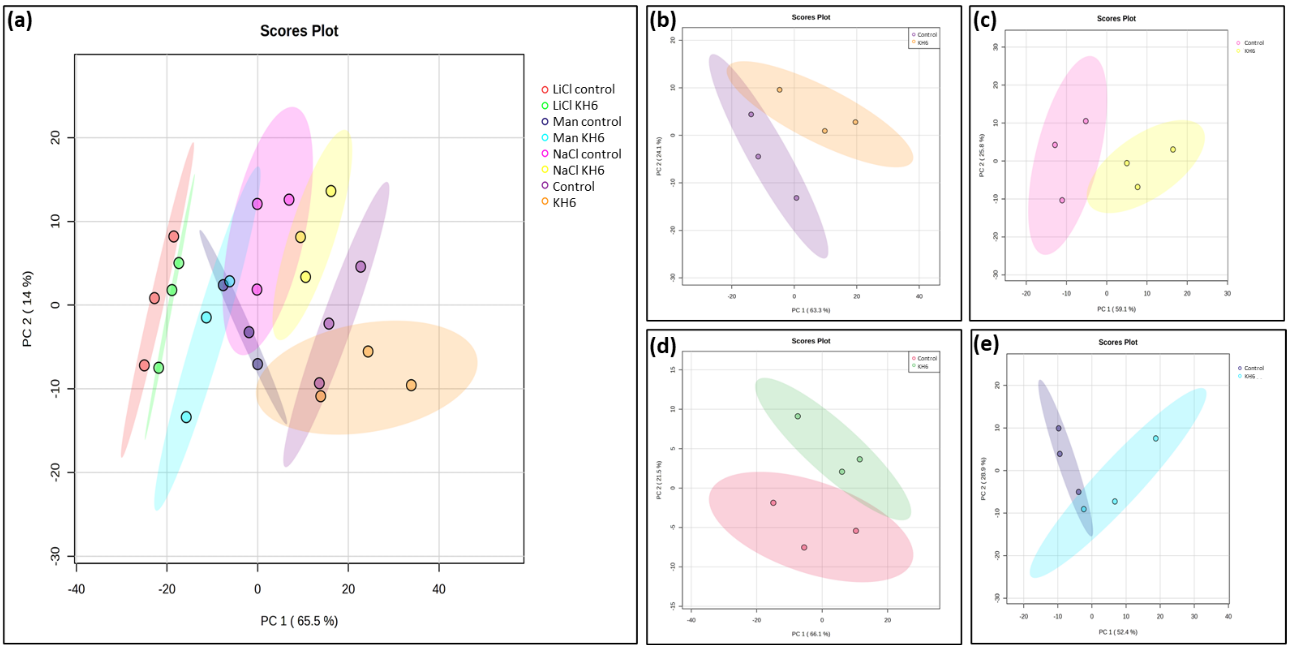

Figure S1. Principal component analysis (PCA) of *A. thaliana* primary metabolites identified by gas chromatography coupled with mass spectrometry (GC-MS). (a) Global difference of the metabolic distribution of all treatments tested (under control, salt (NaCl and LiCl) and osmotic stress (mannitol)) and presence or absence of potassium humate. Different metabolic distribution under (b) control, (c) 140 mM NaCl, (d) 24 mM LiCl and (e) 280 mM mannitol conditions.

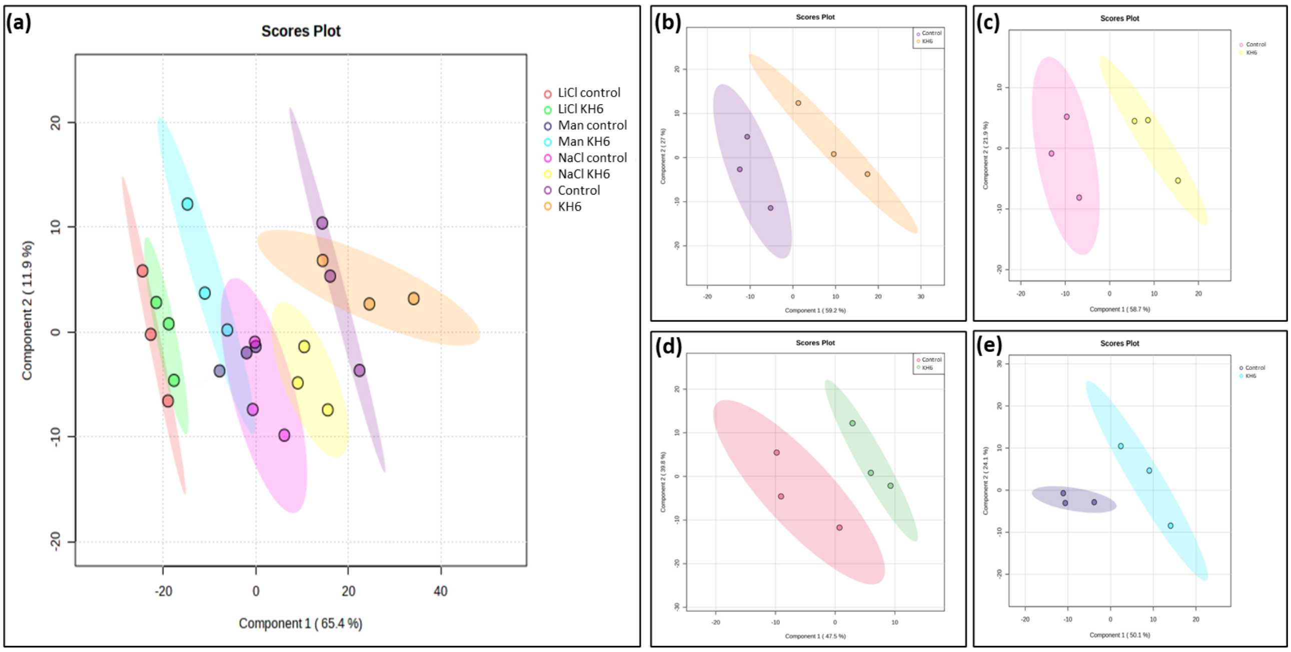

Figure S2. Partial least squares-discriminant analysis (PLS-DA) score plot of *A. thaliana* primary metabolite profiles. (a) Comparative analysis of the four tested conditions (under control, salt (NaCl and LiCl) and osmotic stress (mannitol)) and presence or absence of potassium humate. Different metabolic distribution under (b) control, (c) 140 mM NaCl, (d) 24 mM LiCl and (e) 280 mM mannitol conditions.

Supplementary Table S1. Distribution of the significantly differentially accumulated metabolites between the different conditions tested, represented in the Venn diagrams.

Conditions	Significantly differentially accumulated metabolites
[No stress]	Spermidine, Valine, Serine, Leucine, Sucrose, Malonic acid
[NaCl]	Tartaric acid, GABA, Phenylalanine, Urea, alpha-ketoglutaric acid, Ornithine
[LiCl]	Putrescine, Threonic acid, Glyceric acid
[Mannitol]	Gluconic acid. lactone, Fructose, Glucose
[No stress]<br>[NaCl]	Lysine, Succinic acid, Glycine
[No stress]<br>[Mannitol]	Maleic acid
[NaCl]<br>[LiCl]	Glutamic acid
[NaCl]<br>[Mannitol]	Oxalic acid, Aspartic acid, Fumaric acid, Alanine, Malic acid
[LiCl]<br>[Mannitol]	Proline
[No stress]<br>[NaCl]<br>[LiCl]<br>[Mannitol]	Maltose, Phosphoric acid

Conditions	Significantly up-accumulated metabolites
[No stress]	Serine, Leucine, Sucrose, Malonic acid, Maltose, Glycine
[NaCl]	Tartaric acid, GABA, Phenylalanine, Urea, alpha-ketoglutaric acid, Lysine, Alanine, Ornithine
[LiCl]	Glyceric acid
[Mannitol]	Oxalic acid
[No stress]<br>[NaCl]	Succinic acid
[No stress]<br>[Mannitol]	Maleic acid
[NaCl]<br>[LiCl]	Glutamic acid
[NaCl]<br>[Mannitol]	Aspartic acid, Fumaric acid, Malic acid
[No stress]<br>[LiCl]<br>[Mannitol]	Phosphoric acid

Conditions	Significantly down-accumulated metabolites
[No stress]	Spermidine, Valine ,Lysine
[NaCl]	Phosphoric acid, Glycine, Oxalic acid
[LiCl]	Putrescine, Threonic acid
[Mannitol]	Alanine, Gluconic acid. lactone, Fructose, Glucose
[LiCl]<br>[Mannitol]	Proline
[NaCl]<br>LiCl]<br>[Mannitol]	Maltose
